# Novel approaches to model effects of subconjunctival blebs on flow pressure to improve clinical grading systems after glaucoma drainage surgery

**DOI:** 10.1371/journal.pone.0221715

**Published:** 2019-10-24

**Authors:** Yann Bouremel, Richard M. H. Lee, Ian Eames, Steve Brocchini, Peng Tee Khaw

**Affiliations:** 1 National Institute for Health Research (NIHR) Biomedical Research Centre at Moorfields Eye Hospital NHS Foundation Trust and UCL Institute of Ophthalmology, London, United Kingdom; 2 UCL Department of Mechanical Engineering, London, United Kingdom; 3 UCL School of Pharmacy, London, United Kingdom; 4 Chelsea and Westminster Hospital NHS Foundation Trust, London, United Kingdom; Faculty of Medicine, Cairo University, EGYPT

## Abstract

Clinical grading systems following glaucoma filtration surgery do not include any effects of the bleb on the intra-ocular pressure and are relatively subjective, therefore carrying the risk of inter and/or intra-observer variability. The main objective of the study is to quantify and model the effect of subconjunctival bleb on flow pressure for assessment of clinical grading following glaucoma surgery. Subconjunctival bleb was created by inserting a tube into *ex vivo* rabbit eyes via an *ab externo* approach through the anterior chamber and exiting into the subconjunctival space. Sterile dyed water was injected through the tube into the developing bleb. For the in vitro approach a silicone bleb was created by clamping a circular silicone sheet, injecting dyed water through a fixed resistance outlet tube. Photographic measurements of the bleb height, planform area and pressure were taken as a function of time. Clinical blebs were also collected over a few months. Mathematical algorithm software was used to build the bleb model. Bleb height and volume increase as pressure in the bleb increases. The bleb planform area tended to a constant determined by the section of conjunctiva prior to shunt insertion. These increases were in accordance with the bleb model developed in the Appendix. They show that the pressure in the bleb is related to the resistance of the outflow. The linearity of clinical grading systems is reviewed and a new grading approach is proposed. The pressure in the bleb has a strong dependence on bleb extent, height and a weak dependence on conjunctival thickness. The pressure in a bleb can be estimated from bleb height, radius, and flow rate inlet in agreement with the bleb flow model. These results provide support for an improved bleb categorization system.

## Introduction

Glaucoma is the leading cause of irreversible blindness with estimates suggesting that over 70 million people are affected worldwide, 10% being bilaterally blind [1]. Current glaucoma management plans are aimed at lowering the intraocular pressure (IOP), which is the only proven and treatable risk factor for the disease with several multi-center clinical trials confirming the benefit of lowering IOP in this patient group. Surgical options include glaucoma filtration surgery (GFS) or the insertion of a glaucoma drainage device (GDD). These surgeries shunt aqueous humour from the anterior chamber into the subconjunctival space, resulting in the formation of a small subconjunctival pocket of fluid post-operatively known as a ‘bleb’.

The Ahmed glaucoma device (New World Medical, Rancho Cucamonga, California, USA) has a valve that opens when the IOP reaches a certain level and closes below that pressure, reducing the risk of hypotony. Except for the Ahmed device, most GDDs have a fixed geometry and therefore a fixed flow resistance. For example, the Baerveldt (Abbott Medical Optics, Santa Ana, California, USA) and Molteno implant (Molteno Ophthalmic Limited, Dunedin, New Zealand) GDDs do not contain valves and rely instead on the long-term development of fibrous encapsulation surrounding the bleb to restrict flow. In the early postoperative period, most devices do not give sufficient flow resistance to prevent hypotony with surgeons having to intervene by inserting suture threads into the lumen of the GDD tube. All of these devices drain aqueous humour into the subconjunctival space and result in the formation of a bleb contributing to the patient IOP [2]. Properly “managed” blebs can be the critical difference between success and failure and therefore require close monitoring in the early post-operative period.

**Fig 1:**
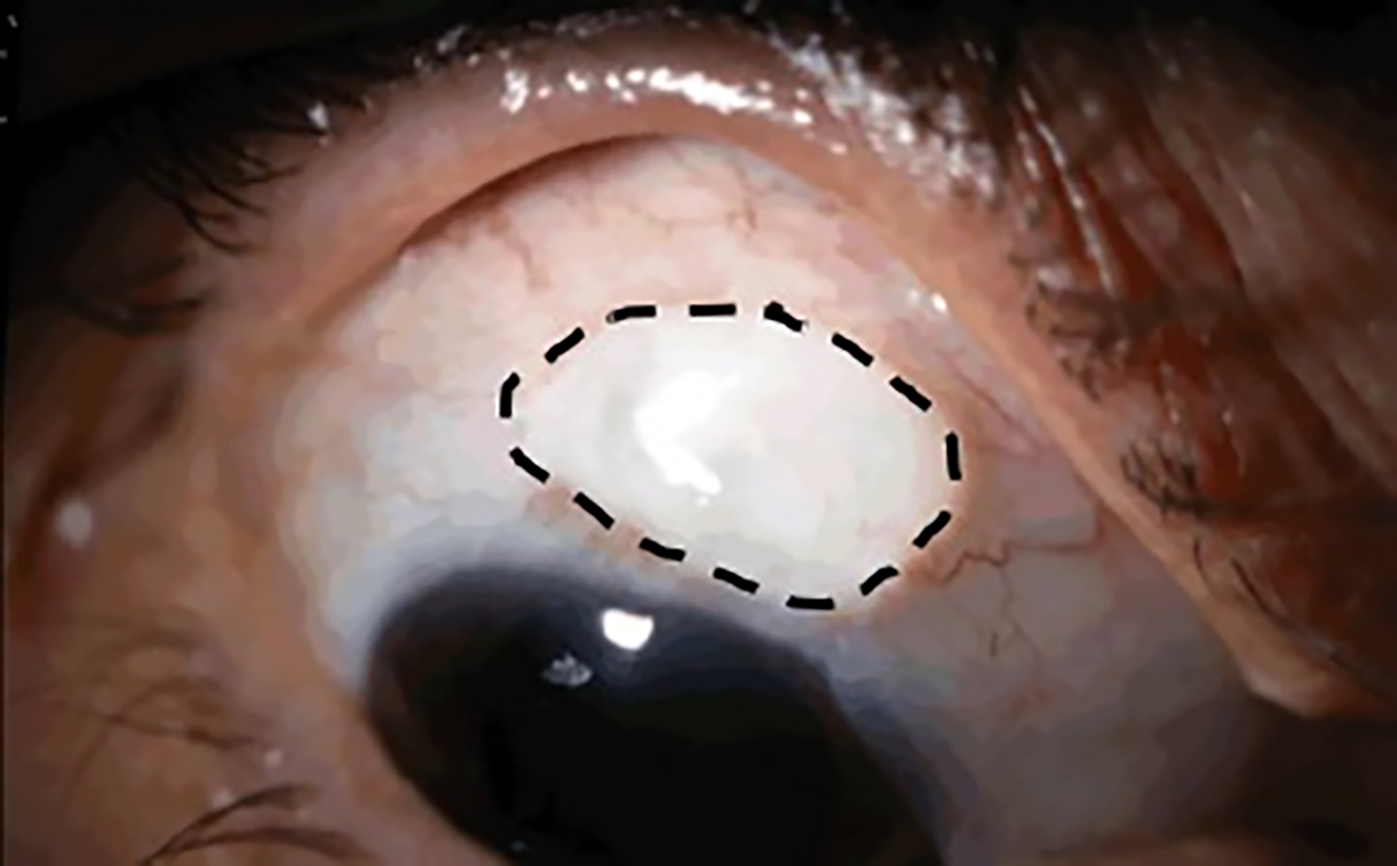
Example of a bleb which, according to MBGS, is graded with a value 3 for height and 50% for extent. The edge of the bleb is indicated by the black dashed line. (Courtesy of MBGS).

Several different grading systems have been proposed to classify blebs and manage their progress: the Indiana Bleb Appearance Grading Scale (IBAGS) [3,4], the Moorfields Bleb Grading System (MBGS, Fig 1) [5], and the “Wuerzburg bleb classification score” (WBCS) [6–8]. These systems are based on a series of parameters that make up to eight categories that include bleb height, extent, vascularity, Seidel test, vessel appearance, encapsulation and microcysts (Table 1). Each category is graded with a score that goes from 0 or 1 up to 5, a higher score usually being associated with a larger value. For example, in the case of the MBGS system and when grading bleb height, a score of 1 is associated with a flat bleb while a score of 4 is considered a high bleb.

**Table 1:** Parameters and scores of the 3 bleb classification systems: Indiana Bleb Appearance Grading Scale, the Moorfields Bleb Grading System and the Picht and Grehn/Wuerzburg bleb classification system.

	Indiana Bleb Appearance Grading Scale (IBAGS) [3,4]	Moorfields Bleb Grading System (MBGS) [5]	Wuerzburg Bleb Classification System (WBSC) [6–8]
Bleb height	H0-H3 (Flat to High)	1–4 (Flat to High)	0–3
Extent	E0-E3 (Small to Large)	Central Area 0%-100%	X
		Maximal 0%-100%	
Vascularity	V0-V4 Avascular Cystic to Extensive vascularity	1–5 (Avascular to Severe)	3–0 (Avascular to Massive)
Seidel test Flow appearance from conjunctiva wound leak	S0 –S2 (No leak to streaming leak)	X	X
Corkscrew vessels	X	X	3–0
Encapsulation	X	X	3–0
Microcysts	X	X	0–3

Fig 1 shows an example of the MBGS grading system with a score of 3 for the bleb height and a score of 50% for the bleb extent.

None of these grading systems includes any effects of the bleb on the IOP and is relatively subjective. This subjectivity may lead to a different grading for the same bleb due to the risk of observer variation, i.e. inter and intra-observer variability.

A few authors have discussed the impact of post-operative blebs on IOP [9–11]. Gardiner *et al*. examined the influence of bleb shape (using a theoretical model) on IOP [11]. The model was based on representing the conjunctiva and sclera as a porous model where the drainage route was through capillaries adjacent to the bleb. While such theoretical models provide some useful indication of the long term influence of bleb shape and scarring on IOP, they do not include the dynamic changes immediately after surgery when the bleb changes shape, and the primary drainage route is towards the back of the eye (into the supra-sclera sub-Tenon’s plane) [11]. Given the importance of IOP measurement on glaucoma surgery outcomes and visual prognosis, we need to understand the effect of a bleb on post-operative IOP.

Further work has been carried out by the authors [12] to show the differences in outflow resistance of bleb obtained using an *ab interno* versus an *ab externo* surgical approach. In this paper, we model the effect of subconjunctival bleb dimensions and conjunctiva properties on flow pressure in a simple geometrical relationship.

## Methods

**Fig 2:**
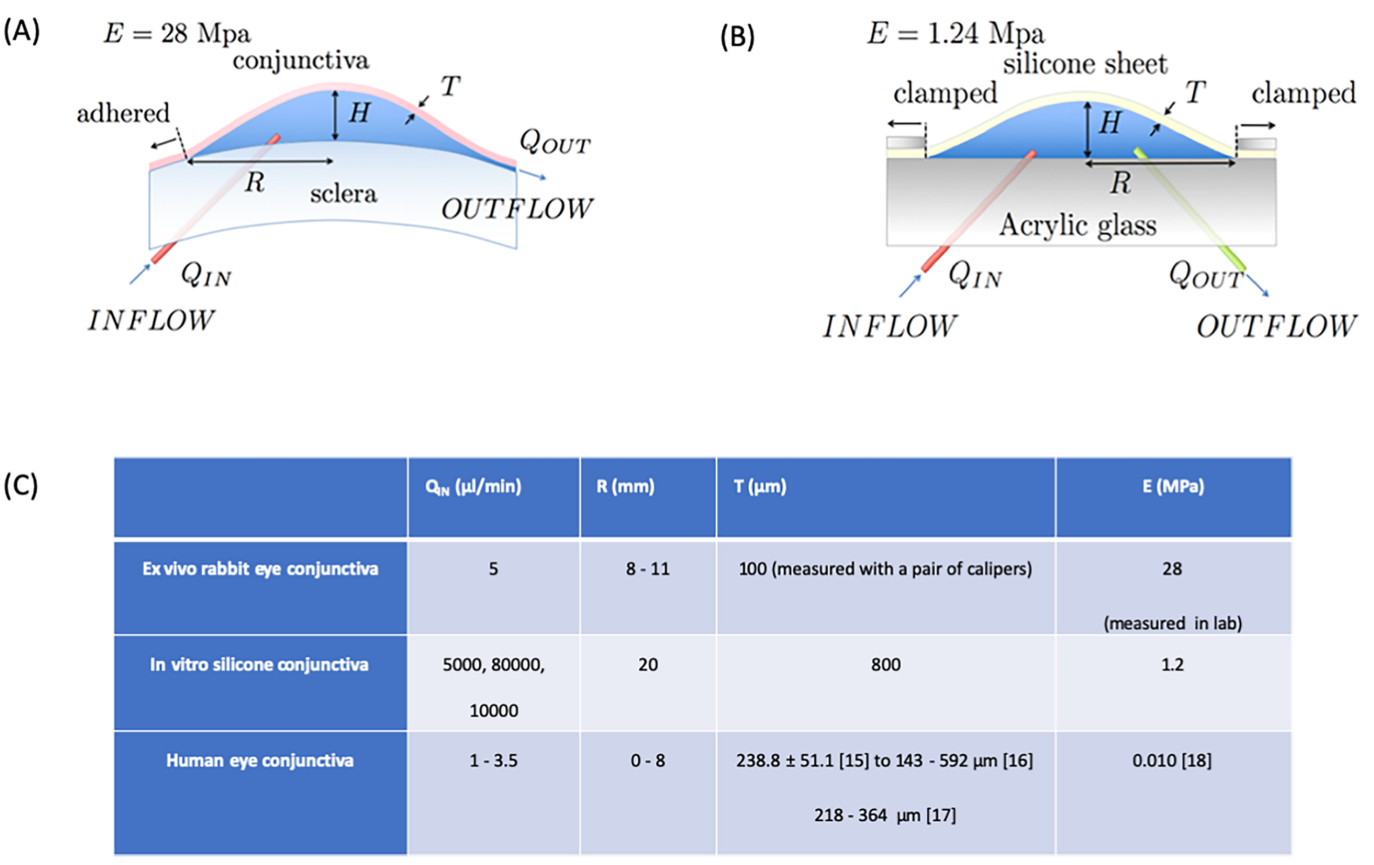
(A, B) Schematics of the ex vivo eye bleb and the in vitro silicone bleb model are shown to highlight the similarities and differences between the two configurations, and (C) table contrasting the physical parameters in the ex vivo, the in vitro silicone model of the bleb and the human. Here *Q*_*IN*_, aqueous flow rate; *R*, bleb radius; *T*, membrane thickness and *E*, Young’s modulus are listed.

Two experimental approaches (*ex vivo* and *in vitro*) were applied here whose configurations are contrasted in Fig 2A and 2B that show the extent (radius *R*), height (*H*) and thickness (*T*) of the blebs with Fig 2C listing the different parameters for the *ex vivo* bleb, the *in vitro* silicone bleb model and the human. In the first case, we created a bleb in an *ex vivo* rabbit eye based on standard operative techniques (Moorfields Safer Surgery System) [13,14]. This technique creates an approximately circular bleb that is fed by aqueous inlet flow *Q*_*IN*_ through a tube, producing a diffuse bleb that is surrounded by conjunctiva with a posterior drainage route. An average bleb radius was reported for each case since the ex-vivo bleb was not perfectly circular. In the second case, a circular silicone sheet was clamped at its edge, pressurized using *Q*_*IN*_. The main difference between the two techniques was the control of the outflow. In the *ex vivo* model, the outflow resistance was due to the circuitous drainage pathway towards the rear of the eye, while in the *in vitro* model the resistance was created (and controlled) by a drainage tube inserted into the bleb.

Both approaches carry advantages and disadvantages. The main benefits of the *in vitro* silicone bleb are that each parameter involved in the bleb volume is controllable, from the resistance outflow to the thickness and the Young’s Modulus of the silicone bleb enabling the derivation of equations. The main disadvantage is the lack of porosity of the silicone bleb or in other terms, the lack of volumetric flux through the tissue. The outflow resistance is, therefore modelled using capillary tubes. However, this disadvantage is solved using the *ex vivo* rabbit eye where the volumetric flux through tissue controls the outflow resistance. In that case, the disadvantage is that the outflow resistance can only be inferred from experiments. Both approaches are complementary to each other with the *in vitro* model enabling the building of the model and the *ex vivo* model, confirmation of the model to real tissue.

A new mathematical bleb model was also developed to interpret and explain these results. This enables the work to be put into a much broader perspective to improve clinical grading systems in the early post-operative period by correlating bleb dimensions to subconjunctival pressure.

The pressure (*P*) in the bleb is correlated to its shape (radius *R*, height *H*), the thickness of the conjunctiva (*T*) and the inlet volume flux *Q*_*IN*_. We describe the methodology based on *ex vivo* and *in vitro* silicone models to measure these quantities.

### Ex vivo model

**Fig 3:**
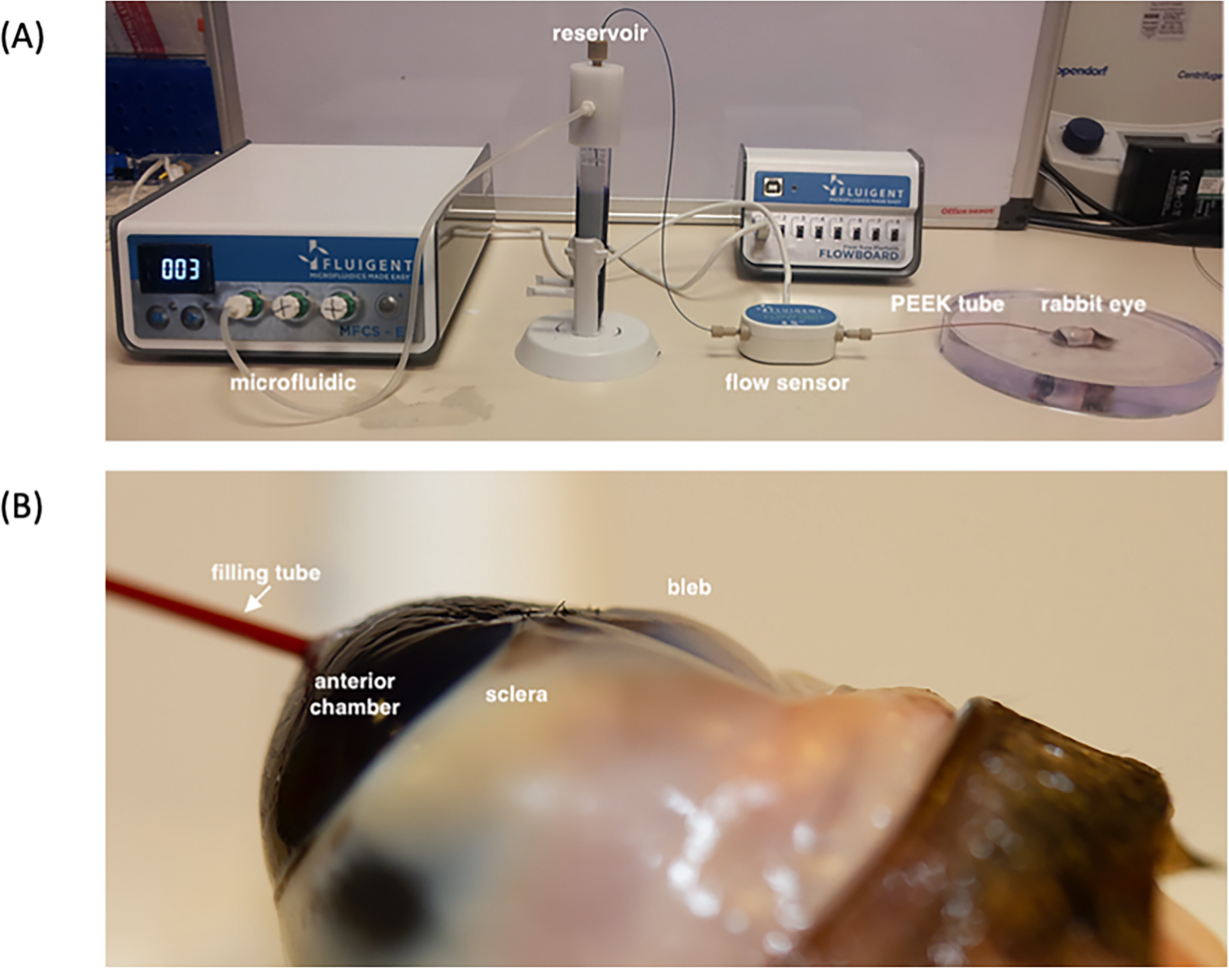
A) photograph showing the experimental set-up. This consists of the microfluidic pump, flow sensor and PEEK tubes connected in series to the rabbit eye. B) Close-up photograph showing the inlet filling tube entering the subconjunctival space via an opening in the cornea and the position of the bleb relative to the sclera and anterior chamber.

The *ex vivo* experiments were conducted at the UCL Institute of Ophthalmology with the experimental setup shown in Fig 3A and 3B). Blebs were prepared using an *ex vivo* rabbit eye via an *ab externo* approach as performed in conventional GFS/GDD surgery. Freshly killed wild rabbit heads were obtained from a local butcher (F Conisbee & Son, Leatherhead, UK). Therefore, there was no need for animal research ethics committee approval. Heads were shipped in a humid container and were approximately 36 hours from death to the start of the experiments. The eye and eyelid margin was exenterated in order to preserve the conjunctival layer as much as possible.

A limbal conjunctival incision was created with Vanna scissors. Blunt dissection with Westcott scissors was performed to the level of the sclera and extended laterally and posteriorly. An 18G (green) cannula was used to create a channel 3mm posterior to the limbus into the anterior chamber, exiting the cornea on the opposite side of the anterior chamber. The cannula needle was exchanged for a 1/32” outer diameter (OD) PEEK tube. The cannula was also removed, leaving the PEEK tube in situ, the sclera forming a surrounding tight seal around the tube with no wound leak. Conjunctival closure was performed with 10/0 nylon (Ethilon, Ethicon, Somerville, NJ, USA). The PEEK tube was connected to a reservoir of a dilute aqueous solution of Coomassie Brilliant Blue G 250 connected to the tube. The blue dye is used as an enhancement method to clearly identify the bleb without affecting the results. The tube and reservoir are connected to a microfluidic pressure pump and flow sensor setup (Fluigent, Villejuif, France). The eye experiments ran for up to 4 hours, and the pressure was recorded at a low frequency of 1 Hz.

A digital SLR camera (Canon EOS 7D) mounted with a 60 mm lens recorded the side view of the eye while another digital SLR camera (Canon EOS 350D) mounted with an EF-S 18-55mm lens was recorded the top view of the eye. Both cameras were controlled through a Wireless Timer Remote Control (Pixel TW-283X) to acquire an image simultaneously every 60 seconds. The bleb dimensions increase with time and the measurements of the bleb height, *H*, and radius, *R*, is estimated by photographic assessment through image processing algorithms written in Matlab.

The aqueous fluid was injected at a rate *Q*_*IN*_ = 5 μl/min into the subconjunctival space forming a bleb between the sclera and conjunctiva. The injection rate affects the time to reach steady state but not the relationship between bleb pressure and height. *H* was measured as the difference in vertical distance of the bleb over time perpendicular to its middle curvilinear point at the start of the experiment. The thickness of the subconjunctival membrane, *T*, was measured using a pair of calipers.

### In vitro model

The *in vitro* model experiments were conducted in the environmental fluid mechanics laboratory located in the UCL Department of Mechanical Engineering with an additional equipment setup.

The conjunctiva layer was modelled as a thin elastic silicone sheet of uniform thickness (*T* = 0.8 mm) sandwiched between a lower clear acrylic plate and an upper steel plate; a circular hole (radius *R* = 20 mm) was cut into the upper plate so that the thin elastic sheet pursed when water was injected beneath the sheet. A PEEK tube was placed below the bleb to act as an outflow with a set resistance of 2.6*10^10^ Pa.s.m^-3^. The inlet flow rate *Q*_*IN*_ was fixed at 5000, 8000 to 10000 μl/min using a syringe pump (PHD 2000 Infusion, Harvard Apparatus, Holliston, United States).

A machine vision camera (Allied Vision Technology) with a 16 mm lens was used to record the side view of the *in vitro* silicone bleb model at a frequency of 1.875 Hz. The bleb height was estimated using calibrated bespoke image processing algorithms written in Matlab similarly to the *ex vivo* approach. A 3-way luer lock connector was used to connect a calibrated pressure transducer to the inlet tube. The pressure signal was converted to volts (P8055-1 Velleman 2003) and recorded at a frequency of 1.875 Hz.

### Mathematical model

A simplified mathematical model was developed to explain the physics of bleb growth and the variation of pressure with time. The model is based on three key processes:

1. Conservation of mass that tells us that the bleb volume grows in time due to the difference between the outflow from the bleb (*Q*_*OUT*_) and the inflow (*Q*_*IN*_).
2. There is a resistance to the outflow of fluid that because of the dominance of viscous effects gives rise to a pressure drop that is proportional to the outflow (*Q*_*OUT*_).
3. The tissue stretches to accommodate the change in bleb volume (*V*) giving rise to a tension in the conjunctiva layer. The pressure in the water within the bleb is obtained from the bleb volume.

The combination of these assumptions can be used to formulate a system of equations that can be solved exactly.

## Results

### Observations

**Fig 4:**
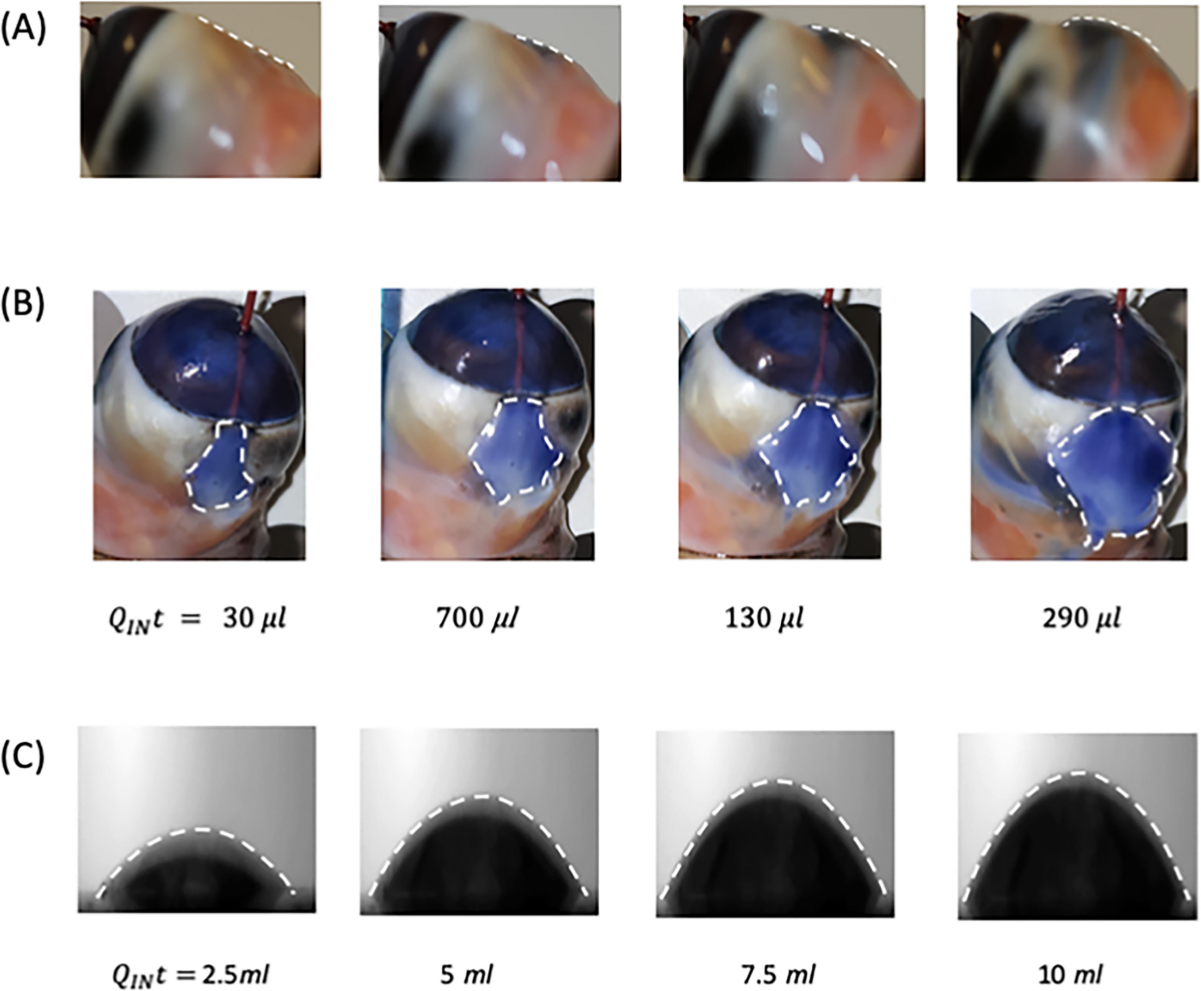
(A), (B) show a photographic sequence indicating the growth in bleb height in an *ex vivo* rabbit eye. The side (top row) and plan (middle row) views are indicated. The border of the dyed (blue) aqueous solution in the subconjunctival space is indicated with a white dashed curve. (C) sequence showing the growth of the in vitro model (side view) with the surface indicated by a white dashed line. In (A), (B) and (C), the progression of the experiment is indicated by the volume of water injected (*Q*_*IN*_ *t*).

The image sequences in Fig 4 are listed in terms of the volume of water injected into the bleb.

Fig 4A and 4B show the side and top view of the *ex vivo* bleb, respectively, as a function of time. The maximum bleb height is located more towards the bleb centre rather than the outlet of the tube. The dashed white line shows the visible extent of the blue dye in the top view of the bleb. The peripheral edge of the bleb appeared to be fixed by the limbal conjunctival incision operated at the beginning of the experiments because the pressure within the sub-conjunctiva space is not enough to peel the borders of the limbal conjunctival incision.

Fig 4C shows a sequence of side images of a circular silicone bleb from the *in vitro* model. The initial bleb height initially increased in proportion to the volume of water injected. The height grew with time and ultimately tended to a steady state shape when a balance between the inflow and outflow was achieved. During the initial filling phase, the bleb heights in Fig 4 increased linearly with time. Fig 4 demonstrates a representative example.

### Height and pressure variation

**Fig 5:**
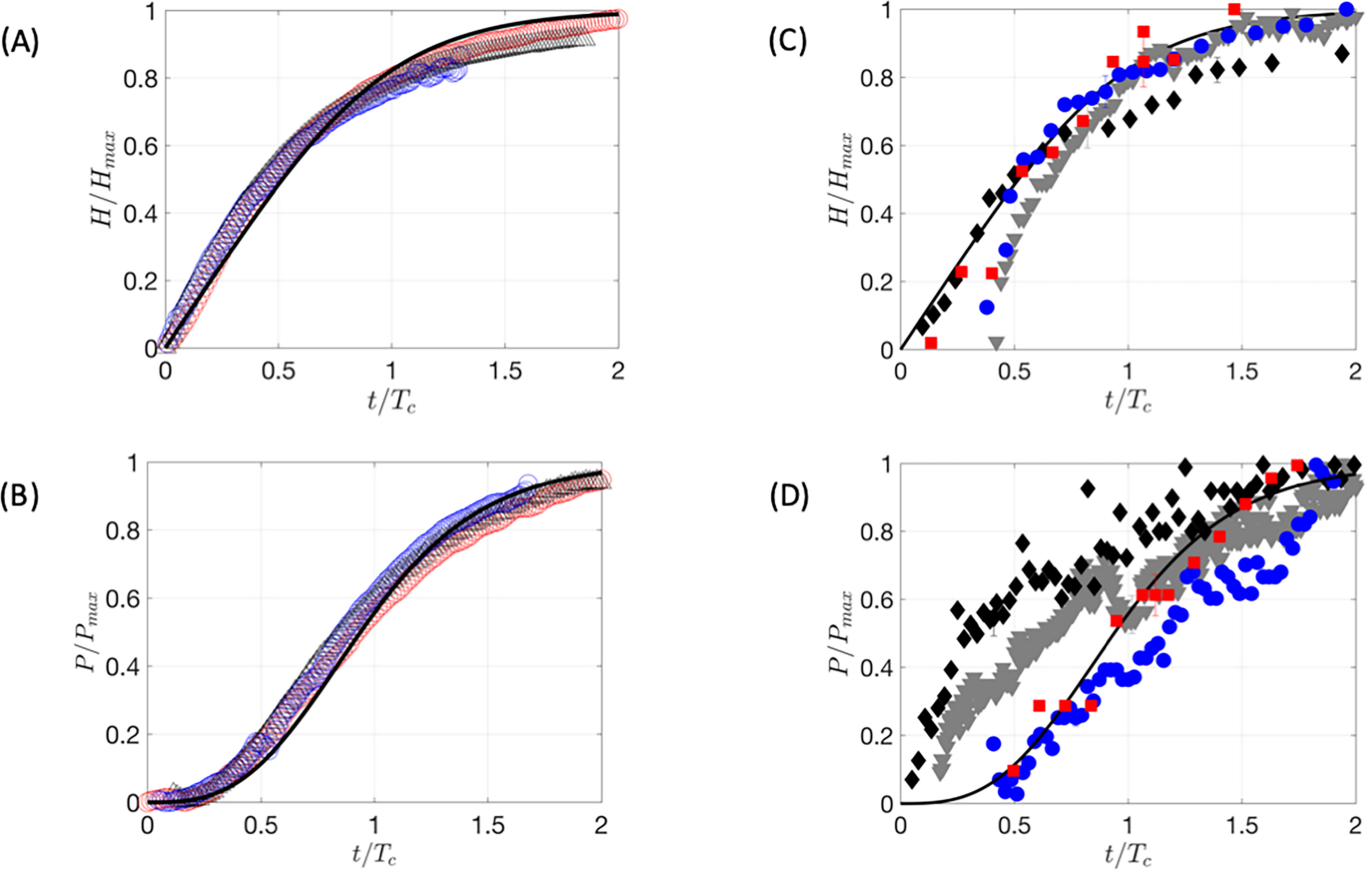
Variation of the in vitro silicone bleb (A) height and (B) pressure in time. The symbols red, blue and black correspond to *Q*_*IN*_ = 5000 μl/min (blue), 8000 μl/min (red), and 10000 μl/min (black). In (C) and (D), the corresponding ex vivo bleb results are plotted. Each symbol corresponds to different ex vivo eyes. The results of the in vitro silicone and ex vivo blebs are compared against the mathematical model developed in the Appendix using a black curve. The curves in (A) and (C) correspond to Eq 11 while the black curves in (B) and (D), to Eqs 11 and 12A.

Fig 5A and 5B show the variation of the *in vitro* bleb height and pressure, respectively, with time. The results from the *in vitro* bleb can be collapsed onto a single (theoretical) curve obtained from the model developed in the Appendix. This enables us to identify the critical height and time taken for the equilibrium to be achieved.

Fig 5A shows that initially the silicone bleb height grew linearly with time because the peripheral edge was fixed and the initial pressure was small because the outflow was low. This is supported by Fig 5B which shows that the pressure was initially small because the silicone bleb contained no water. Both the height and pressure tended to constant values over a characteristic time *T*_*c*_ when a balance between outflow and inflow was achieved.

The development in time is contrasted against the progression of height and pressure for the bleb. The height was observed to vary smoothly and continuously, while increasing monotonically in time. The initial growth of the bleb is slow because it initially contains no water and so the pressure is small. With time, this tends to increase and plateau as the pressure within the bleb equals the pressure given by the outflow *Q*_*OUT*_.

For the *ex vivo* bleb data shown in Fig 5C and 5D, it can be noticed that the height and the pressure of the bleb do not start exactly at zero as a minute volume of fluid was present in the bleb when starting the recording of the experiments due to the surgical operation. While it was possible to start the *in vitro* silicone bleb experiments with no fluid inside the bleb, it was impossible to do so with the *ex vivo* bleb without affecting the conjunctival closure. Therefore, the theoretical model from the Appendix plotted on each graph using a black line was used to estimate the initial relative height and pressure due to the initial minute volume of fluid left by the surgical operation. It can be noted that both graphs follow the theoretical curves for the height in Fig 5C and the pressure in Fig 5D. For the *ex vivo* bleb, no scarring occurs and therefore the plateau characteristics of maximum pressure could not be achieved but estimated from the model.

### Pressure versus height relationship

**Fig 6:**
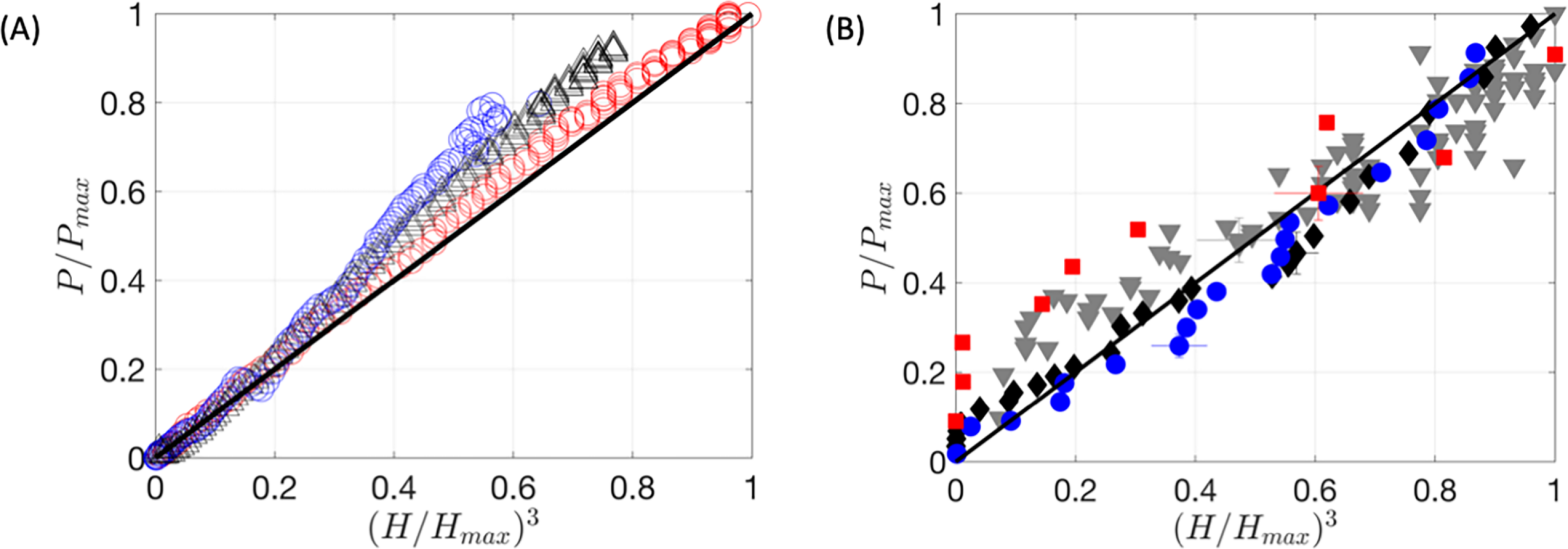
A scatter plot of bleb pressure and height curve is shown for the in vitro silicone bleb in (A) and ex vivo bleb in (B). The symbols used are identical to Fig 5. The results of the in vitro silicone and ex vivo blebs are compared against the mathematical model developed in the Appendix (Eq 12B) plotted with a black curve.

Fig 6A shows a scattered plot of the bleb pressure against bleb height raised to the power of 3. The linearity of this curve is expected for thin elastic sheets and this is confirmed for the *in vitro* model. During this phase, the static pressure forces exerted on the constrained elastic pocket, formed by the stretching of the conjunctiva, is in a quasi-static balance. A similar linearity is observed for the ex vivo bleb in Fig 6B.

## Discussion

### Critical analysis of the experiments and relationship between ex vivo and in vitro

By necessity because of equipment limitations, the flow rate *Q*_*IN*_ into the *ex vivo* bleb and *in vitro* silicone bleb are different to that of the human eye (after surgery). This affects the time *T*_*c*_ taken to reach the maximum bleb height, *H*_*max*_, and the maximum pressure *P*_*max*_. As explained in the Appendix, the bleb characteristics are related through

$$\frac{H_{max}}{R}=0.6{\left(\frac{P_{max}R}{ET}\right)}^{\frac{1}{3}},\frac{Q_{IN}T_{c}}{\pi R^{3}}=\;0.3{\left(\frac{P_{max}R}{ET}\right)}^{\frac{1}{3}}$$ (1)

**Fig 7:**
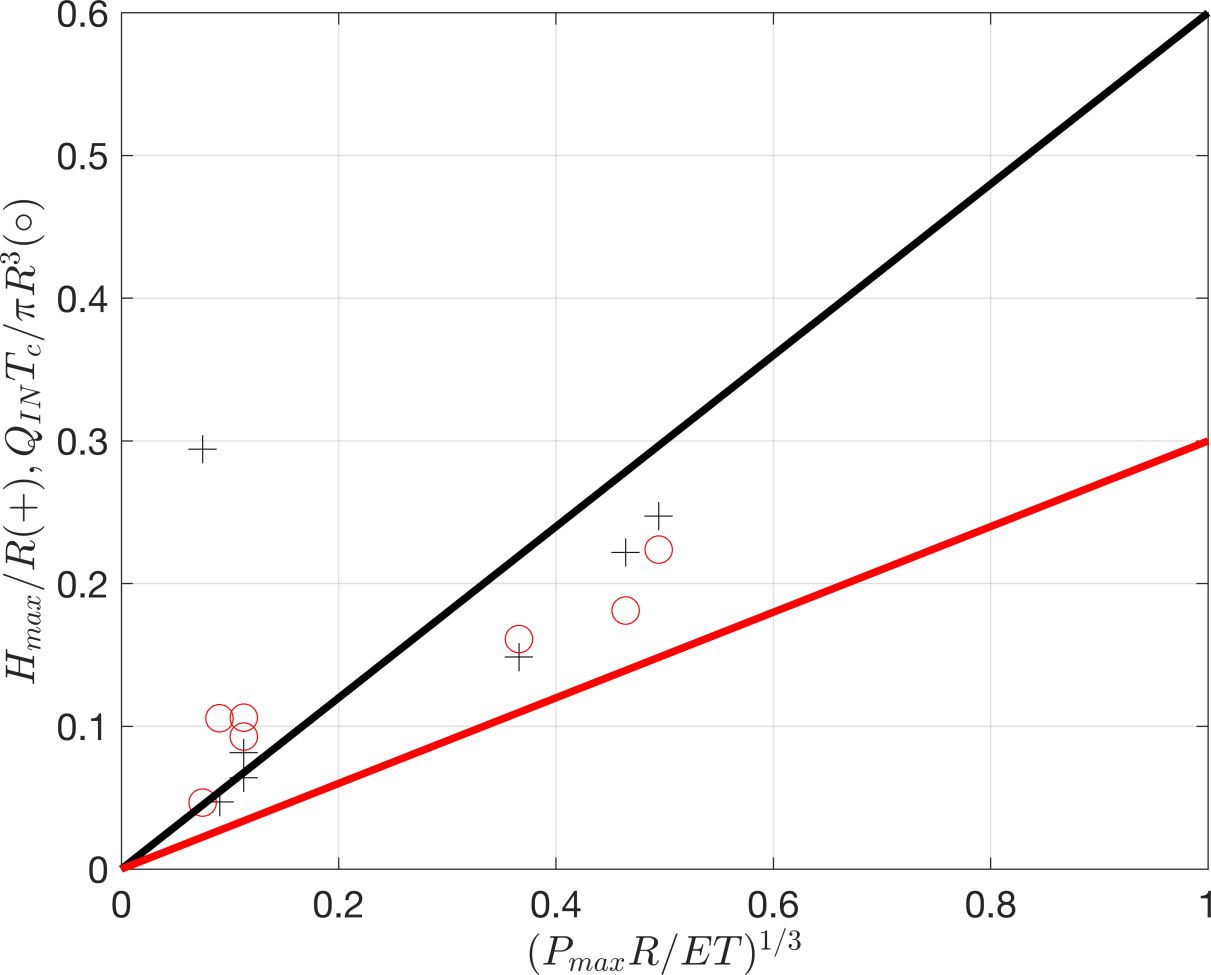
The circles and crosses correspond to H_max_/R and Q_IN_T_c_/πR^3^ respectively in the equilibrium state. The results of the in vitro silicone and ex vivo blebs are compared with Eq 1 plotted with a black curve for H_max_/R and a red curve for Q_IN_T_c_/πR^3^.

Fig 7 shows a synthesis of the maximum height (black crosses) and equilibrium time (red circles) plotted against ${\left(\frac{P_{max}R}{ET}\right)}^{1/3}$. The black and red curves show the predicted relationship of $\frac{H_{max}}{R}$ and $\frac{{Q_{IN}T}_{c}}{\pi R^{3}}$ respectively confirming that the ex vivo and in vitro silicone results fall onto the same trend.

These results can be applied to estimate the response time of a human bleb using typical conditions with $T_{c}=0.5\frac{\pi R^{2}H_{max}}{Q_{IN}}.$ This time period is the ratio of the approximate final bleb volume (~82% of final volume) divided by the filling rate. In the case of a bleb where *P*_*max*_ ≈ 3 mmHg, *H*_*max*_ ≈ 1 mm, *Q*_*IN*_ ≈ 1 μl/min, *R* ≈ 4 mm, *T*_*c*_ ≈ 1509 s. (The typical time taken to equilibrate or the pressure to plateau out would be about 25 to 30 minutes).

### Grading of blebs linearity of scale versus measure of risk

The general purpose of a grading system is to assess risk. The current grading systems are based on subjective estimates of the properties of a bleb. The geometrical measures (lateral extent and height) are linear in the sense that the score increases in proportion to these quantities. The main problem is that the risk that is being measured is best reflected in the intraocular pressure. The two experimental studies of the response of an *ex vivo* bleb and an *in vitro* silicone bleb have shown in Fig 6A and 6B, that the pressure in the bleb (*P*), its height (*H*) and its radius (*R*) are related in a non-linear manner, specifically,

$$P∼\frac{EH^{3}T}{R^{4}}.$$ (2)

The parameters *E* and *T* are respectively the Young’s Modulus and the thickness of the conjunctival layer defined in Fig 2C.

This shows two important points

- The pressure in the bleb (*P*) increases dramatically as the bleb height increases (as confirmed in Fig 6A and 6B and Eq 2).
- The pressure in the bleb decreases dramatically as the extent of the bleb increases (as confirmed by Eq 2).

### Improvements in bleb risk management

**Fig 8:**
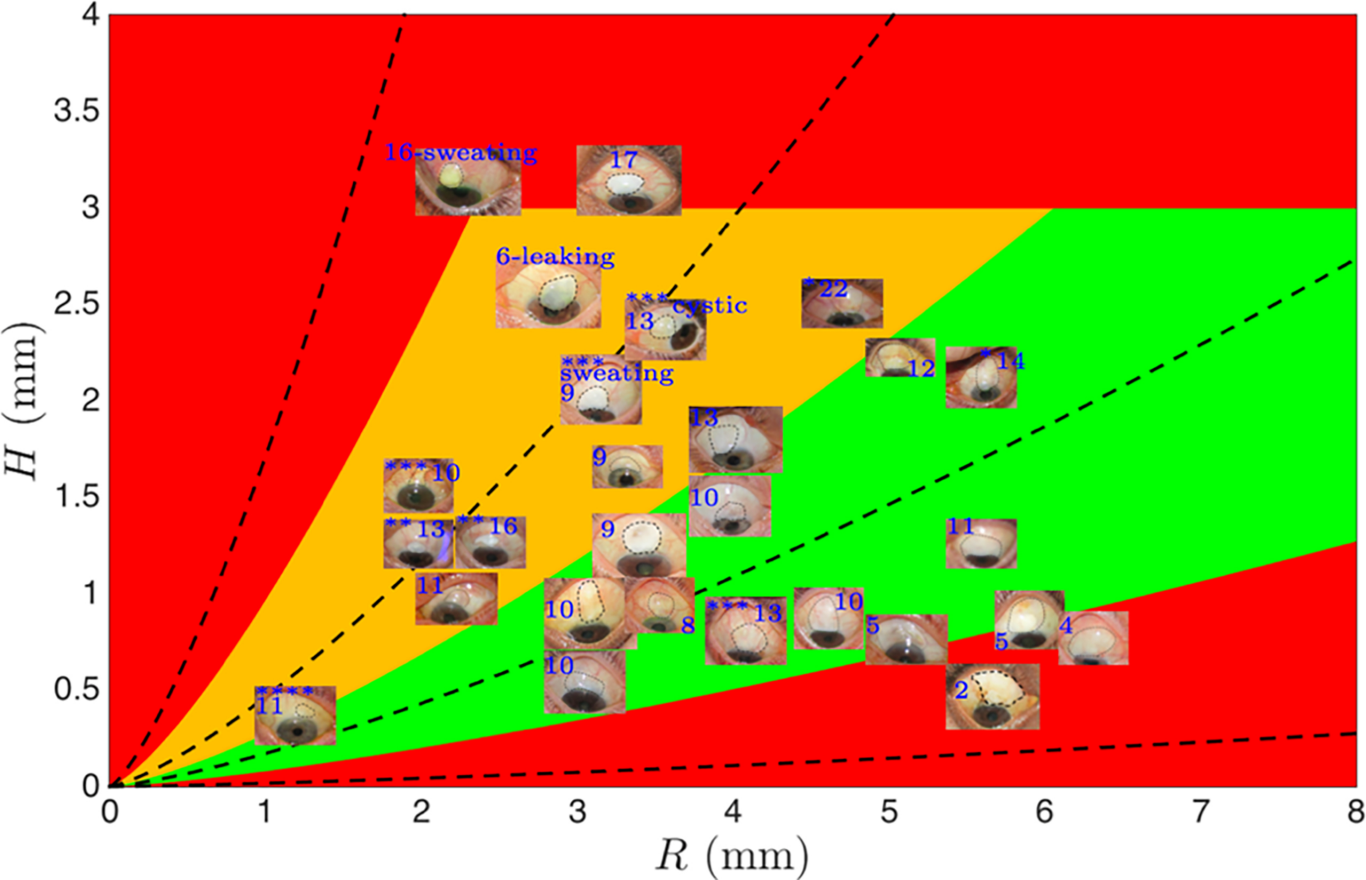
Graphical grading of blebs based on the pressure with green associated to low pressure, orange: Moderate and red: Very high or very low pressure. The parameters are based on geometrical appearance. Photographs from patients are plotted on top of the graphical grading with the IOP indicated in blue and the number of glaucoma drops indicated with stars.

The influence of extent and height of a bleb on pressure are nonlinear and both pieces of information can be combined to estimate pressure. According to Eq 2, it can be seen that a large bleb (high *R*) or a bleb with thin walls (low *T*) or a flat bleb (low *H*) are associated to low pressure while small bleb (low *R*), with thick walls bleb (high *T*) or tall bleb (high *H*) are associated to high pressure. By looking at each of the powers of Eq 2, it can be noticed that each term affecting the pressure is related to different powers. Eq 2 shows that the size of the bleb is extremely important when controlling the pressure, a small bleb is likely to increase the IOP to extremely high values (power of 4). A taller bleb will also be associated to high IOP while the effect is not as dramatic as the size because of the slightly lower power of *H* (power of 3). Finally, the thickness of the bleb plays a lesser role when looking at the pressure as it only appears linearly. The “ring of steel” [19] associated to a small bleb with thick wall will be associated to high IOP. The analysis suggests that more detailed measurements are needed to determine the outcome of surgery and progression of the bleb. The most appropriate route is to measure the extent and height of the bleb and plot them onto the chart in Fig 8. Blebs collected clinically over a few months have been reported on the chart with the corresponding IOP in blue. Iso-pressure contours formed by a bleb of height *H* and radius *R* obtained from Eq 2 are also plotted using black dashed lines. Patient progression parallel to the curves indicates that pressure is not increasing, while progression across these curves indicates that IOP is changing.

To improve the readability, the chart has been divided into three colors:

A red zone that corresponds to three parts:

- the left hand part of the chart associated to small and high blebs denoting high pressure as shown in Eq 2,
- the bottom right hand part of the graph corresponding to large and flat blebs indicating low pressure (Eq 2),
- the top part of the graph related to very tall bleb.

A green zone located in the center that is associated to IOP of approximately 10 mmHg with moderate height and relative large area.

An orange zone that defined a transition zone when going from moderate IOP (green) to high IOP (red).

Each bleb picture is reported with the IOP in blue. When the patient is using glaucoma medication drops, the number of different type of glaucoma drops was added using stars with the number ranging from 1 to 4. If the bleb was sweating or leaking, it was mentioned on the plot. It was decided to calculate the bleb radius to measure the average of the maximum length along the horizontal and the vertical direction and divide it by 2 since most of the blebs were not perfectly circular.

The model has a few limitations that are listed below. When a bleb is leaking or sweating, a relatively large or high bleb can be associated to low IOP since the conjunctiva is leaking and the current purse model is not accurate anymore. Moreover, when patients are using glaucoma drops, the positioning of the blebs on the traffic light plot may not be entirely accurate. Indeed, the intake of drops affects the aqueous humor resistance pathway by decreasing inflow or increasing outflow and therefore the appearance of blebs may not be related to the expected IOP when no drops are used. Finally, when it comes to very small blebs (very low radius and very low height), the model may need to be optimized as no clinical blebs have been obtained yet in this range.

The traffic light plot shows clearly that clinical blebs can be ordered following the purse model based on their dimensions, heights and IOP.

Firstly, blebs located in the red zone correspond to very low (bottom right of the graph) or very high IOP (left and top of the graph).

Secondly, blebs in the green zone are associated to IOP around 10 mmHg without any use of glaucoma drops.

Finally, blebs located in the orange area that should be associated to IOP above 10 mmHg correspond to relatively lower IOP due to the use of glaucoma drops. Indeed, most of the blebs in this area come from patients under glaucoma drops. Therefore, when the IOP increase with the blebs moving from the green to the orange zone, the use of glaucoma drops help to maintain IOP around 10 mmHg.

The traffic light plot shows clearly that bleb sizes are non-linearly correlated to IOP. While very high, very low and/or very small bleb should be avoided according to the traffic light plot, an optimum bleb size may be calculated: large (between 3 and 6 mm radius) with a relative low height (between 0.5 and 1.5 mm). It is also interesting to note that the use of glaucoma drops is able to maintain an acceptable IOP even if the sizes of the blebs demonstrate otherwise.

The current purse model with the traffic light plot is an exciting new method that helps to understand better how blebs and IOP are correlated. This model can also be aimed at the search of the perfect bleb dimensions.

## Conclusions

We studied the development of bleb height and subconjunctival pressure with time due to the constant flow of aqueous to mimic conditions that occur post operatively. Experiments were undertaken using an *ex vivo* and *in vitro* models. We showed that the *ex vivo* and *in vitro* models share similar features, including the relationship between pressure and bleb height. The results show how the filling time, pressure and bleb height are related to aqueous flow rate.

The major outcome of this activity is that immediately post-operation, the bleb pressure can be related to the shape (height, extent) of the bleb. Current bleb grading systems use a linear scale to characterize them. IOP is a proxy measure of the success of the surgical outcome and this is related in a non-linear way to the bleb characteristics. Blebs should be graded in a different manner, for instance, using a chart (Fig 8) that accounts for the non-linear contribution of height and extent to pressure that can be used to map the trend in the bleb development.

## Appendix

The bleb grows due to the difference between the aqueous inflow *Q*_*IN*_ into an elastic pocket and the aqueous outflow through a channel route at a rate *Q*_*OUT*_. The bleb is described as having a planform radius *R* and height *H*, along with pressure *P*. The pressure in the bleb is generated, in this case, by a resistance to the outflow *Q*_*OUT*_.

The mass conservation requires the rate of change of the bleb volume *V* to be due to the differences between the inflow and outflow, so that

$$\frac{dV}{dt}=Q_{IN}-Q_{OUT}.$$ (3)

The bleb is formed from an elastic sheet of thickness *T*, radius *R*, that is characterized by a Young’s modulus *E*. The bleb is assumed to be a spherical cap with a self-similar form

$$V=C_{1}\pi R^{2}H,$$ (4)

where the constant [20] *C*_1_ ≈ 1/2. The flow rate is sufficiently slow that the resistance is proportional to the flow rate through the outflow and

$$P=KQ_{OUT},$$ (5)

where *K* is the resistance of the outflow. Previous works [21] have shown that the pressure in the bleb is related to the bleb height geometry via

$$P=\frac{C_{2}ETH^{3}}{R^{4}},$$ (6)

where the constant *C*_*2*_ = 4.63. For the ex vivo experiments, *E* = 28 MPa and *T* = 0.1mm. For the in vitro experiments, *E* = 1.24 MPa and *T* = 0.8 mm. We have the following ordinary equation

$$C_{1}\pi R^{2}\frac{dH}{dt}=Q_{IN}-\;\frac{C_{2}ET}{KR^{4}}H^{3}.$$ (7)

That can be rewritten with

$$T_{c}=C_{1}\pi R^{2}{\left(\frac{R^{4}K}{C_{2}ETQ_{IN}^{2}}\right)}^{\frac{1}{3}},\;H_{max}={\left(\frac{Q_{IN}R^{4}K}{C_{2}ET}\right)}^{\frac{1}{3}},\;P_{max}=KQ_{IN}.$$ (8)

Writing

$$\tau =\frac{t}{T_{c}},\;\hat{H}=\frac{H}{H_{max}},$$ (9)

reduces the ordinary differential equation to

$$\frac{d\hat{H}}{d\tau }=1-{\hat{H}}^{3}.$$ (10)

This can be integrated to give

$$\tau =\frac{t}{T_{c}}=\frac{1}{\sqrt{3}}\left({\;\text{tan}}^{-1}\left(\frac{\left(2\;\hat{H}\;+1\right)}{\sqrt{3}}\right)-\tan^{-1}\frac{1}{\sqrt{3}}\right)+\frac{1}{6}\log\left(\frac{{\left(\hat{H}+\frac{1}{2}\right)}^{2}+\frac{3}{4}}{{\left(1-\hat{H}\right)}^{2}}\right).$$ (11)

The relationship between pressure and height is

$$\frac{P}{P_{max}}={\left(\frac{H}{H_{max}}\right)}^{3}={\hat{H}}^{3}.$$ (12 a,b)

The in-vitro experimental set-up consisted in a single bleb sheet configuration using three flow rates with identical outflow resistance.
